# Post-Surgical Indications to Radioiodine Treatment and Potential Risk Factors for Post-Treatment Recurrence in Patients with Intermediate-Risk Differentiated Thyroid Carcinoma

**DOI:** 10.3390/jpm13050775

**Published:** 2023-04-29

**Authors:** Mattia Rossi, Chiara Mele, Ruth Rossetto Giaccherino, Letizia Meomartino, Denise Brero, Giulia Marsan, Gianluca Aimaretti, Ezio Ghigo, Loredana Pagano

**Affiliations:** 1 Endocrinology, Diabetology and Metabolism, Department of Medical Sciences, University of Turin, 10126 Turin, Italy; 2 Endocrinology, Department of Translational Medicine, Università del Piemonte Orientale, 28100 Novara, Italy

**Keywords:** thyroid cancer, differentiated thyroid carcinoma, intermediate-risk, radioiodine, recurrence, risk factors

## Abstract

In this multicentric retrospective observational study, we investigated the potential risk factors for radioiodine (RAI) indication and the post-treatment recurrence of intermediate-risk differentiated thyroid cancer (DTC) 1 and 3 years from diagnosis. We included 121 patients who underwent thyroidectomy for intermediate-risk DTC. The 92 patients (76.0%) who underwent RAI treatment had a higher prevalence of extra-thyroid micro-extension (mETE) (*p* = 0.03), pT3 staging (*p* = 0.03) and recourse to therapeutic central (*p* = 0.04) and lateral (*p* = 0.01) neck dissection, as well as higher numbers (*p* = 0.02) and greater dimensions (*p* = 0.01) of lymph node metastases, compared with untreated patients. Relapse was observed in 18.1% and 20.7% of cases 1 and 3 years from diagnosis, respectively, with no significant differences between groups. A lower age at diagnosis (*p* = 0.03) and higher levels of stimulated thyroglobulin (Tg) (*p* = 0.04) emerged as the only independent risk factors for tumour relapse at 1 year. Tumour relapse at 3 years was only independently predicted by the presence of tumour relapse at 1 year (*p* = 0.04). In conclusion, mETE, pT3 and the presence of large, multiple or clinically evident lymph node metastases represent the main indicators for referring patients to RAI treatment. Early recurrence may be considered the most relevant factor when planning further surveillance.

## 1. Introduction

In 2016, the American Thyroid Association (ATA) developed a recurrence-risk stratification which classified differentiated thyroid carcinoma (DTC) into three classes (low-, intermediate- and high-risk) and established a foundational distinction between more-frequent indolent forms and far-less-common relapsing ones.

The recurrence rate of intermediate-risk DTCs ranges between 5% and 20%, and the determination of therapeutic strategy requires further evaluations, specifically regarding the employment of radioiodine (RAI) after surgical treatment.

There is a good deal of agreement about indications to treat in high-risk patients, and stronger evidence has emerged in recent years about the non-inferiority of a follow-up-only strategy for low-risk patients [1]; however, a concrete direction is still lacking for intermediate-risk patients, both in American and more-recent European guidelines [2,3].

Currently, the indication for RAI therapy in intermediate-risk patients is “to be considered”, leaving the final decision to the practitioner, after each patient’s specific details are considered. Regarding such details, greater benefit seems to be obtainable in cases when the major dimension of the tumour is greater than 4 cm [4], in aggressive papillary thyroid cancer (PTC) histology such as tall cell, diffuse sclerosing, and insular variants [5], in patients older than 45 years [6] and in lymph node metastases, especially when the lateral compartment is involved [7]. Another useful tool to guide initial treatment involves testing of baseline and stimulated levels of thyroglobulin (Tg) together with an ultrasound study shortly after surgery; both of these procedures are highlighted in guidelines [2,3], along with the use of later evaluations as an early indicator of relapse [8].

The aim of our study was to investigate the possible indicators that might help clinicians to navigate the debated field of intermediate-risk DTC, when considering indications for radiometabolic therapy. In addition, we sought to investigate predictors for DTC recurrence after follow-up periods of 1 and 3 years.

## 2. Materials and Methods

### 2.1. Patients

This observational retrospective multicentric cohort study included patients who received a histological diagnosis of DTC and met the requirements for “intermediate risk of recurrence”, according to the 2015 ATA guidelines [2], after they were referred to either of two Italian endocrinological centres: the Endocrinology department of the “Azienda Ospedaliera Universitaria—Maggiore della Carità” in Novara and the Endocrinology, Diabetology and Metabolism department of the “Azienda Ospedaliera Universitaria—Città della Salute e della Scienza” in Turin, between 1 January 2017 and 31 December 2020.

### 2.2. Methods

Data were retrieved from electronic hospital records and included the following variables: demographics (age, gender and ethnicity), anthropometrics (weight, height and body mass index—BMI), clinical history and risk factors (smoking habit, radiation exposure, thyroiditis, oncological history, thyroid disease or other cancers in relatives), surgical approach (total thyroidectomy, lobectomy, possible lymphadenectomy of central or lateral neck compartment), histopathological features of the lesion (specific variant, dimensions, side, number of foci, presence of capsular, vascular or extrathyroidal, surgical margins, presence and specifics of metastatic lymph nodes or presence of coexistent thyroiditis), AJCC VIII ed.’s TNM and staging classifications [9], RAI therapy (radioactive dosage, serum-stimulated Tg before RAI, anti-thyroglobulin antibodies (TgAb) or uptake at post-dose whole-body nuclear scan (WBS)), and, finally, 1- and 3-year follow-up (serum Tg, TgAb, ultrasound evidence of pathological tissue or lymph nodes or definition of biochemical or structural recurrence). TgAb positivity was defined as serum TgAb levels above 10 kUI/L: the upper limit for normality of the chemiluminescence microparticle immunoassay (CMIA) test performed in our institutional laboratory (Abbott Ireland Diagnostics. Alinity i Anti-Tg Reagent Kit) Recurrence was defined in accordance with the 2015 ATA dynamic response system [2].

### 2.3. Statistical Analysis

Values were expressed as mean ± standard deviation (SD) or as an absolute number and percentage. Data were tested for normality of distribution with the Shapiro–Wilk test and log-transformed when needed in order to correct for skewness. Mann–Whitney U and chi-square tests were used for comparisons between groups.

Univariate logistic regression analysis was used to evaluate the association between the presence of recurrence (1- and 3-year follow-up) and the following variables: age, sex, clinical and histopathological features of tumour and radioiodine activity.

Multivariate logistic regression analysis was used to identify the potential risk factors for tumour recurrence. The multilinear model included 1- and 3-year tumour recurrence as dependent variables and several independent variables encompassing sex, age, BMI, histopathological features of DTC, radioiodine activity and stimulated Tg values. Odds ratios (ORs), confidence intervals (CIs) and related significance values obtained from the model were reported. A value of *p* < 0.05 was considered as statistically significant. Statistical analyses were performed using SPSS version 21 (IBM Corporation, New York, NY, USA).

## 3. Results

### 3.1. Study Population

We enrolled 121 patients. A summary of the clinical and histopathological features of the study population is presented in Table 1 and Table 2. Of the total population, 74.4% were female subjects, producing a male-to-female ratio of 1:3. Mean age at diagnosis was 48.4 ± 14.9 years (range 18–84 years), and 69.4% of subjects were more than 55 years old. In all, 11.5% of patients had a history of cancer in a different location; the most frequent types were breast cancer in women and prostate cancers in men. A family history of thyroid nodules, autoimmune thyroiditis and thyroid cancer was found in 29.9%, 14.7% and 8.7% of cases, respectively. Familiarity for other malignancies was found in 36.0% of patients.

Most of patients underwent a total thyroidectomy (97.5%), with concomitant prophylactic or therapeutic dissection of the central compartment in 45.5% and 24.0% of cases, respectively. In patients with suggestive ultrasound, a dissection of the lateral neck compartment was also performed (24.0% of cases).

In more than half of the patients, a pathology report proved lymph node metastases (68.4%), primarily in the central compartment (70.1%). The average number of involved lymph nodes was 5.0 ± 4.3, with an average diameter of 10.8 ± 8.8 mm and extracapsular extension in 27.9% of lymph nodes.

The population was then divided into two groups based on whether RAI was performed or not. No significant differences between the two groups emerged in terms of BMI or demographic data. Patients were more frequently referred to RAI if they presented pT3 staging (*p* = 0.03) or extrathyroidal extension of the tumour (*p* = 0.03). In terms of lymph nodes, the number (*p* = 0.02) and size (*p* = 0.01) of lymph node metastases identified at pathological examination both differed significantly between groups, as did the numbers of central (*p* = 0.04) and/or lateral (*p* = 0.01) neck compartment dissections. However, a simple identification of any form of metastasis at the pathological examination did not reveal any statistical significance (*p* = 0.55).

Contrarily, in patients with a history of other malignancies, an indication to RAI was less frequently given by clinicians (*p* = 0.04).

As shown in Table 3, patients undergoing RAI comprised 76.0% of the total population, and the most-used stimulation regimen involved levothyroxine withdrawal (87.8%). The mean level of stimulated Tg was 14.5 ± 45.5 ng/mL, and a minority of patients presented TgAb positivity (15.6%). The majority of patients (76.9%) showed WBS uptake in the thyroid bed, and uptake in the lateral neck compartment or in multiple sites was found in 4.4% of cases.

### 3.2. Follow-Up

Biochemical and ultrasound features of patients at one-year follow-up are summarised in Table 4.

Biochemical evidence of disease was found in eighteen patients (17.1%); however, in only one patient (1.0%) were both biochemical and instrumental evidence of disease documented. This patient had undergone RAI therapy for a classic variant of papillary carcinoma, with extra-thyroid extension and microscopically involved surgical margins.

Patients who underwent RAI generally presented a higher prevalence of tumour relapse, though not to a statistically significant degree. This could be explained by the higher incidence of pT3s, extrathyroidal extension and lymph node metastases at diagnosis.

Follow-up at three years after diagnosis (Table 5) was available for 53 patients. Among these, seven presented biochemical evidence of disease (13.2%), and four presented both biochemical and instrumental evidence of disease (7.5%).

Once again, patients undergoing RAI presented a greater prevalence of relapse.

Conversely, patients who did not undergo RAI were more likely to present thyroidal residual tissue (*p* = 0.01).

### 3.3. Risk Factors for Recurrence

Subsequently, we built two multinomial models to evaluate the association between clinical-histopathological features of DTC and tumour relapse, after controlling for confounders (Table 6). Age at diagnosis (OR = 0.929, IC 95% 0.868–0.994, *p* = 0.03) and stimulated Tg levels (measured at the time of RAI) (OR = 1.015, IC 95% 1.001–1.029, *p* = 0.04) were confirmed to be significant risk factors for one-year tumour recurrence, independently of several confounders, including sex, BMI, histopathological features, metastatic lymph nodes at diagnosis and radioiodine activity. A weak association with the dimensions of the original tumour in terms of greater diameter (mm) was also revealed, though statistical significance was not attained.

With regard to three-year follow-up, tumour relapse was independently predicted only by a previously confirmed relapse at one-year follow-up (OR = 15.414, IC 95% 1.120–212.101, *p* = 0.04).

## 4. Discussion

In this multicentric, observational, retrospective cohort study, we evaluated the main clinical, biochemical and histological features that might guide clinicians towards an indication for RAI treatment in cases of intermediate-risk DTC, as defined by ATA.

We analysed clinical and histological variables for each individual and found that the main indicators considered by our clinicians when referring patients to RAI treatment were as follows: a family history of thyroid cancer, a personal history of tumour in a different location, clinical and histological proof of lymph node metastasis and both the number and dimensions of lymph nodes.

We found that a medical history of cancer reduces the appeal of RAI. This might be explained by the persistent fear of inducing the onset of a second tumour following RAI; because of this, clinicians tend to avoid such treatment in patients with an oncological history. However, it is known that radioiodine activity administered during therapy is not related to the onset of other cancers, as evidenced by the study of Hirsch et al. in which no significant associations were found between RAI and an increased risk of other neoplasms, and the incidence of second cancers was found to be greater in the control (non-RAI) group (9.2% of treated patients vs. 10.5% of non-treated patients) [10]. Similar findings were reported by Bhattacharyya and co-workers [11]. The most frequently reported tumours in our study were prostate cancer in men and breast cancer in women. The onset of non-metastatic breast cancer in RAI-treated patients is still a matter of debate; however, and in line with other neoplasms, RAI does not appear to increase its risk, as Zhang et al. confirmed [12]. Rather, an RAI-independent two-way association seems to exist between breast cancer and DTC itself; i.e., women with a previous history of breast cancer are at greater risk of onset of DTC and vice versa [13].

One controversial finding in our study is the significantly higher incidence of a family history of DTC in the group of patients who were not sent to RAI treatment. This appears to be in contradiction with established evidence that familiar forms are associated with more aggressive clinical presentation (i.e., multicentric, bilateral or invasive presentation). Still, strong evidence is lacking in the literature regarding the association between histological features and poorer prognoses and whether family history is per se a recurrence-predictive factor when considered independently [14]. It may be that our clinicians did not primarily consider family history when evaluating indication for RAI but instead focused on more histological factors which could have acted as confounders.

In our experience, the therapeutic dissection of central and lateral neck compartments and both the number and dimensions of involved nodes were positively associated with RAI application. Interestingly, this finding could not be obtained by mere identification of any form of lymph node metastasis at pathology examination. In our opinion, this apparent contradiction can be explained in terms of clinically significant lymph node metastasis: not all metastases, but only large ones, multiple ones or ones that prompted the surgeon towards neck dissection drove the indication to RAI.

The debate remains open, and ATA guidelines themselves suggest considering treatment, even if conflicting observational data support disease-free survival [2]. However, stronger evidence is available concerning RAI employment when specific features of disease are considered. For instance, in a similar population, Lee et al. [15] showed that lymph node metastases reduced recurrence-free survival rates. Seong Young Kwon et al. [16] showed that a high dose of ^131^I is necessary to prevent recurrence when more than five nodes are involved. Finally, Han et al. [17] suggested that RAI should not be employed in cases of intermediate-risk DTC with central lymph node involvement, especially if the number of lymph nodes involved is less than five.

From a histological point of view, pT3 staging and mETE are significantly associated with a greater inclination towards RAI. However, even in these cases, evidence is substantially composed of observational data, which is sometimes conflicting, and RAI is only generally suggested. The results of our study conflict with more recent findings in the literature. Indeed, mETE has been associated with a slightly higher risk of recurrence [18], but this increase seems to be clinically irrelevant [19], and several authors have suggested that these tumours should be considered as low risk [20]. In a recent Italian observation-based study of a population with of low-to-intermediate risk DTCs, no differences were found in the response rates of patients with or without mETE [21]. Moreover, in other studies, mETE was ruled out as an independent predictive factor for response to RAI, especially in cases of pT1–pT2 staging and of local metastasis-free lesions [22,23,24].

A secondary purpose of our investigation was to deduce possible independent predictors of tumour relapse at one and/or three years.

Among the investigated variables, a lower age at diagnosis was found to be significantly predictive for disease recurrence at one-year follow-up: the earlier the onset of the tumour, the higher the probability of relapse one year after treatment. This outcome seems to be in open contradiction with established findings in the literature which consider the advancing of age as a negative predictive factor for tumour response. However, this issue is still the subject of live debate; indeed, in a recent work by Forleo et al., age at diagnosis was not revealed to be a factor that significantly impacts the outcome of intermediate-risk disease at one-year follow-up [21]. Still, an older age at diagnosis does appear to significantly raise the burden of mortality, as reported by a number of authors, including Kim et al., 2016 and Yan et al., 2018 and recently acknowledged by the AJCC in its VIII ed. of its TNM classification system, which set 55 years as a new cut-off for Stage I DTC, independent from pT and pN factors [9,25,26]. Regarding follicular histotype, though, mortality does appear to drastically increase at ages above 45 years. Because of this, patients aged between 45 and 54 years could be inappropriately placed in a lower stage and be negatively impacted by such an underestimation [26].

In our series, the activity of administered ^131^I (100 mCi = 3.7 GBq) was not associated with relapse one year after treatment. This finding has been largely confirmed in recent studies which have shown no adjunctive effect of a high activity (e.g., 3.7 GBq) versus a lower one (e.g., 1.1 GBq) [27,28,29], particularly in the follicular variant of papillary thyroid carcinoma [30].

Pre-ablation-stimulated Tg is another independent predictive factor for the evidence of relapse at one-year follow-up. Supporting data for this are readily available in the literature, both in older studies which confirm pre-ablation-stimulated Tg combined with TgAb, post-ablation Tg and ATA risk, as an efficient tool in predict one-year relapse [31], and more recent research, which has associated the first post-surgery Tg detection with definite predictions of persistence and one-year recurrence, particularly in papillary microcarcinomas [32].

Finally, we tried to unravel possible factors influencing a longer follow-up (three years). Only one-year relapse resulted in a higher risk of three-year relapse, suggesting the need for a closer follow-up for such patients. Hence, the need for more studies with longer observational periods.

We believe the main strength of our study relies in its specific evaluation of intermediate-risk patients, a group for which evidence is lacking in the literature and which requires greater agreement in terms of clinical courses of action. Another strength of our study derives from its long follow-up period of three years. The main limitations are related to the observational and retrospective nature of our study.

## 5. Conclusions

In conclusion, as regards our primary endpoint (i.e., possible post-treatment indications to post-surgery RAI therapy), we may state the following: a higher prevalence of mETE, pT3 staging and large or multiple clinically evident lymph node metastases all result in a higher appeal to RAI.

Moreover, as regards our secondary endpoint (i.e., possible predictors for disease recurrence): one-year response appears to be best predicted by a lower age at diagnosis and a higher pre-RAI stimulated Tg level. One-year response was itself the only independent risk factor for three-year recurrence; consequently, early recurrence might be considered the most relevant factor when planning further surveillance.

## Figures and Tables

**Table 1:** Clinical features of the population as a whole and subdivided into two groups, based on whether or not RAI therapy was performed.

Variables		Whole Population (n = 121) N (%)	RAI (n = 92)	Non-RAI (n = 29)	*p*-Value
**Sex, N (%)**	Male	31 (25.6)	25 (27.2)	6 (20.7)	0.48
	Female	90 (74.4)	67 (72.8)	23 (79.3)	
**Age at diagnosis (years) (mean ± SD)**		48.4 ± 14.9	47.5 ± 15.0	51.2 ± 14.3	0.24
**Ethnic group, N (%)**	Caucasians	115 (95.0)	87 (94.6)	28 (96.6)	0.67
	Hispanic	3 (2.5)	3 (3.3)	0 (0.0)	0.32
	Asian	2 (1.7)	1 (1.1)	1 (3.4)	0.38
	African	1 (0.8)	1 (1.1)	0 (0.0)	0.57
**BMI (kg/m^2^) (mean ± SD)**		25.5 ± 4.9	25.3 ± 4.8	26.3 ± 4.9	0.34
**Risk factors, N (%)**	Previous radiation (available for 114 pts) ^1^	10 (8.8)	7 (7.6)	3 (10.7)	0.68
	Thyroiditis (available for 115 pts) ^1^	16 (13.9)	14 (15.2)	2 (7.1)	0.23
	Smoke (available for 116 pts) ^1^	21 (18.1)	16 (18.2)	5 (17.9)	0.51
	Tumours of other origin (available for 113 pts) ^1^	13 (11.5)	7 (7.6)	6 (22.2)	**0.04**
**Family history, N (%)**	Nodules (available for 117 pts) ^1^	35 (29.9)	25 (27.2)	10 (35.7)	0.44
	Thyroid Carcinoma (available for 115 pts) ^1^	10 (8.7)	5 (5.4)	5 (17.9)	**0.04**
	Thyroiditis (available for 116 pts) ^1^	17 (14.7)	13 (14.1)	4 (14.3)	0.95
	Tumours of other origin (available for 114 pts) ^1^	41 (36.0)	28 (30.4)	13 (48.1)	0.13
**Surgery, N (%)**	Lobectomy	3 (2.5)	1 (1.1)	2 (6.9)	0.08
	Total Thyroidectomy	118 (97.5)	91 (98.9)	27 (93.1)	
	Prophylactic CC Dissection	55 (45.5)	43 (46.7)	12 (41.4)	0.61
	Therapeutic CC Dissection	29 (24.0)	26 (28.3)	3 (10.3)	**0.04**
	LC Dissection	29 (24.0)	27 (29.3)	2 (6.9)	**0.01**

*p*-values for Mann–Whitney or χ2 tests are shown. SD—standard deviation; BMI—body mass index; pts—patients; RAI—radioiodine therapy; CC—neck central compartment; LC—neck lateral compartment. ^1^ Due to the retrospective nature of the study it was not possible to recover anamnestic details from a small number of patients who did not provide contact details. *p*-Values highlighted with **bold**, reached statistical significance (<0.05).

**Table 2:** Histopathological features of the population as a whole and subdivided into two groups based on whether or not RAI therapy was performed.

Variables		Whole Population (n = 121) N (%)	RAI (n = 92)	Non-RAI (n = 29)	*p*-Value
**Lymph node MTS ^1^**	Any lymph node involvement N (%), (available for 98 pts) ^2^	67 (68.4)	58 (74.4)	9 (45.0)	0.55
	Number of involved lymph nodes (mean ± SD), (available for 67 pts) ^3^	5.0 ± 4.3	5.5 ± 4.5	1.8 ± 0.7	**0.02**
	Dimension (mm) (mean ± SD), (available for 67 pts) ^3^	10.8 ± 8.8	12.2 ± 8.4	0.6 ± 0.7	**0.01**
	Capsular invasion, N (%), (available for 67 pts) ^3^	12 (27.9)	11 (28.9)	1 (20.0)	0.67
**Histological features, N (%)**	Bilateral	25 (20.7)	20 (21.7)	5 (17.2)	0.60
	Multifocal	46 (38.0)	37 (40.2)	9 (31.0)	0.37
	mETE	58 (47.9)	49 (53.3)	9 (31.0)	**0.03**
	Vascular invasion	63 (52.1)	51 (55.4)	12 (41.4)	0.20
**Margins, N (%)**	R0	96 (85.7)	70 (83.3)	26 (92.9)	0.21
	R1	15 (13.4)	1 (0.9)	13 (15.5)	0.26
	R2	1 (1.2)	2 (7.1)	0 (0.0)	0.56
**Staging, N (%)**	T1a	20 (16.7)	12 (13.0)	8 (28.6)	0.07
	T1b	39 (32.5)	29 (31.5)	10 (35.7)	0.68
	T2	24 (20.0)	18 (19.6)	6 (21.4)	0.83
	T3	37 (30.8)	33 (35.9)	4 (14.3)	**0.03**
	N0	17 (14.0)	11 (12.0)	6 (20.7)	0.24
	N1a	42 (34.7)	34 (37.0)	8 (27.6)	0.35
	N1b	23 (19.0)	22 (23.9)	1 (3.4)	**0.01**
	Nx	39 (32.2)	25 (27.2)	14 (48.3)	**0.03**

*p*-values for Mann–Whitney or χ^2^ tests are shown. SD—standard deviation; mETE—micro extra-thyroidal extension; MTS—metastases; pts—patients; RAI—radioiodine therapy. ^1^ Evidence and details of lymph node metastases were recovered from pathology reports. ^2^ Only 98 patients underwent lymph node dissection (central compartment ± lateral compartment) which allowed the identification of lymph node metastases. ^3^ Details were only available for patients with established lymph node metastasis. *p*-Values highlighted with **bold**, reached statistical significance (<0.05).

**Table 3:** Summary of RAI treatment characteristics and related investigations.

Variable		RAI (N = 92, 76.0%)
**RAI activity (mCi) ^1^, (mean ± SD)**		83.9 ± 23.4
**Stimulation regimen, N (%)**	LT4-withdrawal	79 (87.8)
	rhTSH	11 (12.2)
**Stimulated Tg (ng/dL), (mean ± SD)**		14.5 ± 45.5
**TgAb, N (%)**	Elevated ^2^	14 (15.6)
	Negative	76 (84.4)
**Uptake at WBS, N (%)**	No uptake	13 (14.3)
	Thyroid bed	70 (76.9)
	Neck LC	4 (4.4)
	Mediastinum or Lungs	0 (0.0)
	Bone	0 (0.0)
	Multiple locations	4 (4.4)

RAI—radioiodine therapy; LT4—levothyroxine; rhTSH—recombinant thyrotropin; Tg—thyroglobulin; TgAb—anti-thyroglobulin antibodies; WBS—post-RAI whole-body scan; LC—neck lateral compartment. ^1^ 100 mCi = 3.7 GBq. ^2^ TgAb was defined as “elevated” when >10 kUI/L, the upper limit of normality of our kit.

**Table 4:** Biochemical and ultrasound features at 1-year follow-up in the population as a whole and subdivided into two groups based on whether or not RAI treatment was performed.

1-Year Followup (Available for 107 pts) ^1^				
Variables		RAI (n = 86) N (%)	Non-RAI (n = 21) N (%)	*p*-Value
**TgAb**	Elevated ^2^	8 (9.3)	0 (0.0)	0.35
	Negative	78 (90.7)	21 (100.0)	
**Basal Tg (mean ± SD) (ng/mL)**		1.6 ± 6.2	0.3 ± 0.5	0.32
**Stimulated Tg (mean ± SD) (ng/mL)**		25.4 ± 94.9	-	-
**Thyroid ultrasound**	Residual tissue	19 (22.1)	4 (19.0)	0.76
	Suspicious tissue	0 (0.0)	0 (0.0)	-
	Suspicious lymph nodes	3 (3.5)	0 (0.0)	0.38
**Recurrence ^3^**	No recurrence	65 (78.3)	21 (95.5)	0.06
	Biochemical recurrence	17 (20.5)	1 (4.5)	0.08
	Structural and biochemical recurrence	1 (1.2)	0 (0.0)	0.60

*p*-values for Mann–Whitney or χ^2^ tests are shown. RAI—radioiodine therapy; SD—standard deviation; Tg—thyroglobulin; TgAb—anti-thyroglobulin antibodies. ^1^ Available for patients who reached 1-year follow-up at the moment of data gathering. ^2^ TgAb was defined as “elevated” when >10 kUI/L, the upper limit of normality of our kit. ^3^ Recurrence was defined in accordance with the 2015 ATA dynamic response system [2].

**Table 5:** Biochemical and ultrasound features at 3-year follow-up in the population as a whole and subdivided into two groups based on whether or not RAI treatment was performed.

3-Year Followup (Available for 53 pts) ^1^				
Variables		RAI (n = 42) N (%)	Non-RAI (n = 11) N (%)	*p*-Value
**TgAb**	Elevated ^2^	3 (7.1)	0 (0.0)	0.36
	Negative	39 (92.9)	11 (100.0)	
**Basal Tg (mean ± SD) (ng/mL)**		0.9 ± 3.3	0.6 ± 0.6	0.75
**Stimulated Tg (mean ± SD) (ng/mL)**		15.9 ± 13.1	-	-
**Thyroid ultrasound**	Residual tissue	4 (9.5)	5 (45.5)	**0.01**
	Suspicious tissue	0 (0.0)	1 (9.1)	0.06
	Suspicious lymph nodes	4 (9.5)	0 (0.0)	0.29
**Recurrence ^3^**	No recurrence	32 (76.2)	10 (90.9)	0.28
	Biochemical recurrence	7 (16.7)	0 (0.0)	0.15
	Structural and biochemical recurrence	3 (7.1)	1 (9.1)	0.83

*p*-values for Mann–Whitney or χ^2^ tests are shown. RAI—radioiodine therapy; SD—standard deviation; Tg—thyroglobulin; TgAb—anti-thyroglobulin antibodies. ^1^ Available for patients who reached 3-year follow-up at the moment of data gathering. ^2^ TgAb was defined as “elevated” when >10 kUI/L, the upper limit of normality of our kit. ^3^ Recurrence was defined in accordance with the 2015 ATA dynamic response system [2]. *p*-Values highlighted with **bold**, reached statistical significance (<0.05).

**Table 6:** Multinomial logistic regression models for recurrence at 1 and 3 years of surveillance.

**1-YEAR FOLLOW-UP ^1^ Recurrence (No = 0, Yes = 1)**	**Variables**	**OR**	**IC 95%**	***p*-Value**
	Sex	0.822	0.121–5.574	0.84
	**Age at diagnosis (years)**	**0.929**	**0.868–0.994**	**0.03**
	BMI (kg/m^2^)	1.173	0.973–1.416	0.09
	Papillary tumour	2.829	0.365–21.908	0.32
	Tumour dimension (mm)	0.918	0.835–1.010	0.08
	mETE	1.058	0.222–5.043	0.94
	Presence of lymph node MTS ^2^	1.970	0.213–18.223	0.55
	Radioiodine activity (mCi) ^3^	1.007	0.974–1.042	0.67
	**Stimulated Tg (ng/mL)**	**1.015**	**1.001–1.029**	**0.04**
**3-YEAR FOLLOW-UP ^1^ Recurrence (No = 0, Yes = 1)**	Sex	0.321	0.016–6.327	0.45
	Age at diagnosis (years)	0.964	0.880–1.057	0.44
	BMI (kg/m^2^)	1.246	0.903–1.721	0.18
	Papillary tumour	2.836	0.167–48.284	0.47
	Tumour dimension (mm)	0.989	0.839–1.167	0.90
	mETE	0.170	0.008–3.513	0.25
	Presence of lymph node MTS ^2^	1.021	0.027–38.756	0.99
	Radioiodine activity (mCi) ^3^	1.047	0.976–1.124	0.20
	**Stimulated Tg (ng/mL)**	**15.414**	**1.120–212.101**	**0.04**

Tg—thyroglobulin; TgAb—anti-thyroglobulin antibodies; BMI—body mass index; mETE—micro extra-thyroidal extension; MTS—metastases. ^1^ Binary variables: sex, papillary tumour, mETE, presence of lymph node MTS. Continuous variables: age at diagnosis; BMI; tumour dimensions, radioiodine activity, stimulated Tg. ^2^ Presence of lymph node metastases at pathology report, in any form (binary variable). ^3^ 100 mCi = 3.7 GBq. Features that reached statistical significance (*p*-Value < 0.05) are highlighted in bold.

## Data Availability

Data are not included in any public repository but are freely available for sharing. Please refer to the corresponding author for more information.
